# Foot Posture Outweighs Habitual Footwear in Discriminating Concurrent Lower-Limb Pain in Children: A Cross-Sectional Interpretable Machine-Learning Study

**DOI:** 10.3390/life16101639

**Published:** 2026-09-30

**Authors:** Hakan Büyükçelebi, Mahmut Açak, Özgür Eken, Abdulkadir Atalan, Alperen Şanal, Ebru Yalçın, Hadeel R. Bakhsh, Monira I. Aldhahi

**Affiliations:** 1 Department of Coaching Education, Faculty of Sport Sciences, Inonu University, Malatya 44000, Türkiye; hakan.buyukcelebi@inonu.edu.tr; 2 Department of Coaching Education, Faculty of Sport Sciences, Canakkale Onsekiz Mart University, Canakkale 17100, Türkiye; m.acak@hotmail.com (M.A.); alperensanal48@gmail.com (A.Ş.); yalcinebru17@gmail.com (E.Y.); 3 Department of Physical Education and Sport Teaching, Faculty of Sport Sciences, Inonu University, Malatya 44280, Türkiye; ozgur.eken@inonu.edu.tr; 4 Department of Industrial Engineering, Faculty of Engineering, Canakkale Onsekiz Mart University, Canakkale 17100, Türkiye; abdulkadiratalan@gmail.com; 5 Department of Rehabilitation Sciences, College of Health and Rehabilitation Sciences, Princess Nourah bint Abdulrahman University, P.O. Box 84428, Riyadh 11671, Saudi Arabia; hrbakhsh@pnu.edu.sa

**Keywords:** paediatric lower-limb pain, foot pain, flatfoot, foot posture, footwear, machine learning, explainable artificial intelligence, SHAP, diagnostic prediction model

## Abstract

Lower-limb pain is common in school-aged children, but the relative contributions of foot posture, adiposity, and footwear remain uncertain, and prediction models may be inflated by target leakage. We aimed to quantify how well foot posture, adiposity, and footwear discriminate current lower-limb pain, and whether machine learning outperforms penalised logistic regression. We analysed a cross-sectional school-based dataset of 4408 children aged 4–14 years from Türkiye. Eight classifiers were trained with five-fold cross-validation and evaluated once in a held-out 20% test set; pain-site information was excluded from the primary predictors because it encoded the outcome. Test values of the area under the receiver operating characteristic curve (AUROC) ranged from 0.945 to 0.962; extremely randomised trees (0.961; 95% CI 0.942–0.977) did not outperform penalised logistic regression (ΔAUROC 0.0003, 95% CI −0.0075 to 0.0095; *p* = 0.99). Foot posture alone reached an AUROC of 0.909, whereas demographics plus anthropometry reached 0.704, and adding footwear increased this only to 0.723. Therefore, concurrent lower-limb pain was discriminated mainly by foot posture and adiposity, while footwear and algorithmic complexity added little. As posture and pain were recorded concurrently, the models discriminate current rather than future pain. The findings are most relevant to first-contact assessment in general practice.

## 1. Introduction

Foot and lower-limb complaints are among the most frequent musculoskeletal reasons for which children are brought to primary care, and paediatric flatfoot in particular generates a disproportionate share of specialist referrals despite being, in most children, a physiological and self-limiting variant [1,2]. The clinical difficulty is not detecting a low medial longitudinal arch but deciding which foot will hurt. Most flexible flat feet are asymptomatic and require no intervention, whereas rigid deformity, restricted subtalar motion, or an underlying coalition is far more likely to be painful and to warrant investigation [1,3,4,5,6]. Because the definition of paediatric flatfoot is heterogeneous and observer-dependent [7], efforts to identify the child at risk of symptoms have relied on relatively coarse clinical descriptors. Clinical, radiographic, and gait-based investigations further demonstrate substantial heterogeneity in paediatric flatfoot phenotype and function [8,9,10].

Two candidate explanations dominate parental and professional discourse. The first is body mass. Overweight and obese children display flatter footprints, thicker medial midfoot fat pads, higher midfoot contact area, and higher plantar pressures than their leaner peers [11,12,13,14,15]; higher plantar loading in overweight children is associated with reduced physical activity [16]. Obese children also show higher plantar pressure and lower foot sensitivity than non-obese peers [17]. Excess body mass may also act through calf-muscle flexibility: limited ankle dorsiflexion is more frequent in overweight and obese schoolchildren [18,19], and tightness of the gastrocnemius or gastrocnemius–soleus complex is a frequently overlooked contributor to symptoms, because a flexible flatfoot with a short Achilles tendon, unlike the simple flexible flatfoot, is known to cause pain and disability in some adolescents [20,21]. Static and dynamic foot characteristics and arch loading vary substantially across childhood [22,23]. The second explanation is footwear. Habitual footwear alters spatiotemporal, kinematic, and electromyographic gait parameters in children [24]; incorrectly fitted footwear is associated with foot pain and foot disorders across the lifespan [25]; early comparative work reported a higher prevalence of flat foot among habitually shod children [26]; and foot-strike patterns differ between habitually barefoot and habitually shod youth [27]. Therapeutic-footwear research in children remains heterogeneous [28]. In many middle-income settings, a third, rarely studied dimension is added: the authenticity and construction quality of the shoe, since counterfeit and unbranded footwear is widely worn and may differ systematically in sole stiffness, heel-counter rigidity, and last geometry from the branded product it imitates. Whether such shoe-level attributes translate into measurable differences in symptom prevalence, once child-level factors are accounted for, is unknown.

Radiographic assessment of flatfoot can show interobserver variability [29]. Machine learning (ML) has been adopted rapidly in this field, mostly for the automated recognition of foot type from plantar-pressure maps, radiographs or footprint images, where reported accuracies frequently exceed 0.95 [30,31,32,33,34,35]. Comparable claims are now appearing for tabular, questionnaire-based paediatric foot datasets. Such figures deserve scrutiny. TRIPOD+AI and PROBAST+AI provide current reporting and risk-of-bias guidance for prediction models using regression or artificial intelligence [36,37]. These frameworks distinguish diagnostic models, which estimate the probability that a condition is present at the time of assessment, from prognostic models, which estimate the risk of a future event [38]; cross-sectional data can support only the former. Calibration is essential for clinically interpretable risk estimates [39], and systematic evidence shows no consistent performance advantage of machine learning over well-specified logistic regression in low-dimensional clinical prediction settings [40]. Adequate sample-size planning is likewise central to prediction-model development [41]. Cross-sectional foot surveys almost always record the anatomical site of pain alongside the presence of pain, and the “no pain” category of the site variable is a deterministic function of the outcome; including it as a predictor guarantees near-perfect discrimination while conveying no clinical information. This is a textbook instance of target leakage, a failure mode documented across scientific fields [42].

Therefore, we set out to answer three questions in a large school-based paediatric sample. First, how accurately can concurrent lower-limb pain be discriminated from routinely observable child characteristics, foot posture, and footwear attributes when outcome-adjacent variables are removed? Second, which predictor blocks actually carry that information—anthropometry, footwear, or clinical foot posture? Third, does algorithmic complexity, including a deep neural network, buy anything over regression? We additionally quantify, within the same data, the magnitude of the inflation produced by the leakage described above, so that the resulting benchmark can be interpreted by future investigators working with similar datasets.

## 2. Materials and Methods

### 2.1. Study Design, Setting, and Participants

This is a secondary analysis of a cross-sectional, school-based dataset of 4432 children aged 4 to 14 years in Türkiye. Children were recruited from multiple private nurseries, day-care centres, kindergartens, primary schools and secondary schools in the city centre and the Ezine district of Çanakkale; recruitment was school-based with predefined eligibility criteria rather than random sampling. Children were not included if they had a foot condition that could interfere with assessment (e.g., oedema, ulcers or wounds), a history of foot fracture leading to deformity, a major foot deformity or amputation, or a neurological disorder (e.g., cerebral palsy, spina bifida or Down syndrome). Height was measured barefoot to the nearest millimetre with a portable calibrated stadiometer (Seca 213, Seca GmbH & Co. KG, Hamburg, Germany). and body mass to the nearest 0.05 kg with a calibrated electronic scale (Tanita BC-420 SMA, Tanita Corporation, Tokyo, Japan). The exact number of participating institutions and children’s school membership were not retained in the de-identified analytic dataset, so clustering of children within schools could not be modelled (Section 4.5). Each record contained sex, age (completed years), standing height (cm), body mass (kg), body mass index (BMI, kg·m^−2^), examiner-classified foot posture, habitual footwear type, footwear quality, self-reported lower-limb pain status and, for symptomatic children, the anatomical site of pain. The study was approved by the Health Sciences Ethics Committee of Çanakkale Onsekiz Mart University (Decision No. 10/13; Project No. 2024-YÖNP-5358; approval date: 10 December 2024) and was conducted in accordance with the Declaration of Helsinki; written informed consent was obtained from the parents or legal guardians of all participants. The descriptive component is reported in accordance with STROBE and the prediction-model component in accordance with the TRIPOD+AI statement [36]; the completed checklists are provided in Supplementary Tables S1 and S2.

### 2.2. Variables

Foot posture was recorded in four mutually exclusive clinical categories: normal, flexible (reducible) flatfoot, rigid flatfoot, and high-arched (cavus) foot. Foot posture was assessed in the nursery or school setting from static footprints obtained with a podoscope (Chinesport S.p.a., Udine, Italy): after the plantar surfaces had been cleaned with alcohol and dried, each child stood barefoot with body weight evenly distributed on both feet, still for at least 5 s and looking straight ahead, and the images were analysed with the Global Postural System/PODATA software(version 5,Udine, Italy). The Staheli Arch Index (SAI; narrowest width of the arch region divided by the widest width of the heel print) was used to classify the arch [43]: SAI ≤ 0.29, high-arched (cavus) foot; 0.30–0.70, normal; 0.71–1.00, flexible flatfoot; ≥1.01, rigid flatfoot. The Jack test (passive dorsiflexion of the hallux in neutral stance) [44] was additionally used to distinguish flexible from rigid flatfoot, with reconstitution of the medial longitudinal arch indicating flexibility. Classifications were reviewed by two field experts (both experienced physiotherapists with expertise in paediatric foot-posture assessment), and disagreements were resolved by consensus; no valid inter-rater reliability coefficient is available for this dataset. The assessors were blinded to pain status and pain location. Only the resulting categories, not the continuous SAI values, were retained in the analytic dataset. Footwear type comprised eight habitual categories: canvas high-top, leather high-top flat-soled, soft canvas flat, soft leather high-top, medium-stiff leather with a thick sole, stiff leather flat, closed-toe slipper, and sandal. Footwear quality comprised three categories: branded original, counterfeit branded, and unbranded. The outcome was the binary presence of self-reported lower-limb pain: children were asked whether they experienced pain in their feet or legs after sports activities or prolonged walking, and the site of pain was recorded for those who answered yes. The site of pain (posterior leg; posterior leg and medial foot; anterior leg; plantar foot; ankle; no pain) was retained for descriptive purposes and for a secondary multiclass analysis but was excluded from every predictor set in the primary analysis for the leakage reason set out above. Because the question covered both the feet and the legs, and the recorded pain sites extend from the leg to the foot, the outcome is referred to as lower-limb pain rather than foot pain. The sites were combined in the primary analysis because non-specific leg pain in children, particularly posterior-leg (calf) pain, was clinically linked to foot posture [45], although a controlled comparison did not confirm a meaningful association [46], and because three of the five sites had only 27–45 cases, too few for stable site-specific multivariable models; grouped region-specific analyses are described in Section 2.6.

### 2.3. Data Quality Procedures

Three pre-specified cleaning rules were applied before any modelling. Records containing codes outside the defined coding frame were removed (*n* = 5). Records in which pain status and pain site were logically inconsistent—pain reported with a “no pain” site, or no pain reported with a specified painful site—were removed (*n* = 19), since these could not be resolved without access to the original forms. BMI values were recomputed from height and mass and agreed with the recorded values to within 0.006 kg·m^−2^ for every record. Twenty children had biologically implausible BMI values (<11 or >35 kg·m^−2^); these were retained in the primary analysis and removed in a sensitivity analysis. Because BMI in kg·m^−2^ varies with age and sex in children, BMI was additionally expressed as a BMI-for-age z-score (BAZ), using the WHO Child Growth Standards for children younger than 5 years [47] and the WHO growth reference for school-aged children and adolescents for those aged 5 years or older [48]; because age was recorded in completed years, the mid-point of each year of age was used. BAZ values below −5 or above +5 were flagged as biologically implausible [49]. The analytic sample was therefore 4408 children. No values were missing for any variable.

### 2.4. Statistical Analysis

Continuous characteristics are summarised as mean ± standard deviation and compared between children with and without lower-limb pain using Welch *t*-tests, with Mann–Whitney *U* tests as a distribution-free check and Cohen *d* as the effect size. Normality was not tested formally to choose between tests, because with groups of 716 and 3692 children such tests flag trivial departures from normality, whereas the Welch *t*-test remains valid for skewed data in samples of this size [50]; the Mann–Whitney *U* test served as a confirmatory check. Categorical characteristics are compared using the χ^2^ test with Cramér’s *V*. A multivariable logistic regression model containing age, BMI, sex, foot posture, footwear type and footwear quality was fitted to the complete analytic sample to obtain adjusted odds ratios (ORs) with 95% Wald CIs; height and mass were omitted from this model in favour of BMI to avoid collinearity. Because some predictor categories had very high or very low outcome prevalence, which can inflate maximum-likelihood odds ratios through sparse-data bias [51], the model was refitted by Firth’s penalised likelihood [52,53] with profile-penalised likelihood CIs. Because the outcome was common (16.2%), ORs overstate the corresponding risk ratios [54]; crude prevalence ratios (PRs) were therefore also reported. In a further sensitivity model, BMI was replaced by BAZ; linearity of its effect was examined with a restricted cubic spline and effect modification by age with a BAZ × age interaction term. Two-sided *p* < 0.05 was considered significant.

### 2.5. Prediction Modelling

Because predictors and outcome were ascertained at the same examination, all models are diagnostic (concurrent) prediction models in the TRIPOD+AI sense [36,38]: they estimate the probability that a child currently reports lower-limb pain, not the risk of future pain, and “prediction” is used here in this statistical sense only. The analytic sample was split into a training partition (80%, *n* = 3526) and a held-out test partition (20%, *n* = 882), stratified on the outcome, with a fixed random seed. All preprocessing—standardisation of continuous variables and one-hot encoding of categorical variables—was implemented inside cross-validation pipelines and fitted on training folds only, so that no information from validation or test data entered any transformation [42].

Eight classifiers were compared: L2-penalised logistic regression, k-nearest neighbours, a support vector machine with a radial basis function kernel, random forest [55], extremely randomised trees, XGBoost [56], LightGBM [57] and a feed-forward deep neural network (multilayer perceptron with up to four hidden layers, ReLU activations, Adam optimisation and early stopping). Hyperparameters were selected by grid search maximising the area under the receiver operating characteristic curve (AUROC) in five-fold stratified cross-validation of the training partition. Class imbalance (16.2% events) was handled by class-weighting rather than synthetic oversampling, so that no synthetic observations could cross the train–test boundary. The decision threshold for each model was fixed a priori from the training data as the value maximising the Youden index of the out-of-fold cross-validated predictions, and was then applied unchanged to the test partition.

The held-out partition was used once. Discrimination is reported as AUROC and as the area under the precision–recall curve (AUPRC); threshold-dependent performance is reported as accuracy, balanced accuracy, sensitivity, specificity, positive and negative predictive value (PPV, NPV), F1 and the Matthews correlation coefficient (MCC); probabilistic accuracy is reported as the Brier score. CIs and between-model differences in AUROC were obtained from 2000 bootstrap resamples of the test partition. Calibration was assessed graphically, by the Brier score, by the Hosmer–Lemeshow statistic, and by the calibration intercept (calibration-in-the-large) and calibration slope [58], before and after isotonic recalibration of the best model fitted within the training partition [39]. The same calibration measures were computed for penalised logistic regression.

Model behaviour was interpreted in two complementary ways. Permutation importance was computed on the test partition (30 repetitions, AUROC as the scoring function) for the best model, and SHapley Additive exPlanations (SHAP) values were computed with a tree explainer for the XGBoost model, which shares the same feature space [59]. To quantify where the predictive information resides, four nested models were fitted with a common gradient-boosting learner: (A) demographics and anthropometry; (B) A plus footwear type and quality; (C) B plus foot posture (the full model); and (D) foot posture alone.

### 2.6. Sensitivity and Secondary Analysis Methods

Three additional analyses were pre-specified. First, the full pipeline was repeated after excluding the 20 children with implausible BMI values. Second, to quantify the effect of the leakage discussed in the Introduction, a model was deliberately fitted with the pain-site variable included as a predictor. Third, among symptomatic children only, a five-class model of the anatomical site of pain was fitted and evaluated by five-fold cross-validation against the majority-class baseline using accuracy, macro-averaged F1, and Cohen κ. Four additional analyses were conducted post hoc in response to peer-review comments. First, region-specific models were developed by restricting the outcome to either pain involving the foot or ankle—defined as plantar foot, ankle, or posterior leg pain with medial foot involvement (*n* = 229)—or pain confined to the posterior or anterior leg (*n* = 487). Each group was compared with the 3692 pain-free children. The original training/test allocation was retained after excluding children with pain at other anatomical sites. A histogram-based gradient-boosting classifier with default settings and a multivariable logistic regression model were applied. Second, Firth-penalized logistic regression and crude prevalence ratios were calculated, as described in Section 2.4. Third, BMI was replaced with BAZ in the primary model. Fourth, the calibration intercept and slope were estimated as described in Section 2.5.

### 2.7. Software

Analyses were performed in Python 3.12 using scikit-learn 1.8 [60], XGBoost 3.4 [56], LightGBM 4.7 [57], SHAP 0.52 [59], statsmodels 0.14, and SciPy 1.17. Analysis code is available from the corresponding author. BMI-for-age z-scores were computed with rcpchgrowth 4.6 (WHO references), and Firth-penalised logistic models were fitted with purpose-written code maximising the penalised likelihood, with profile-penalised likelihood confidence intervals.

## 3. Results

### 3.1. Sample Characteristics

Of 4432 records, 24 (0.5%) were removed by the pre-specified cleaning rules, leaving 4408 children (2179 boys, 49.4%; mean age 9.51 ± 2.83 years; mean BMI 18.24 ± 4.32 kg·m^−2^). Lower-limb pain was reported by 716 children, a prevalence of 16.2% (95% CI 15.2–17.3%). Among symptomatic children, the most frequent site was the posterior leg (*n* = 442, 61.7%), followed by the posterior leg together with the medial foot (*n* = 158, 22.1%), anterior leg (*n* = 45, 6.3%), plantar foot (*n* = 44, 6.1%) and ankle (*n* = 27, 3.8%). Pain therefore involved the foot or ankle in 229 children (32.0%) and was confined to the leg in 487 (68.0%). Mean BAZ was 0.22 ± 1.52, and 8 children had BAZ values outside ±5.

Table 1 shows the univariable comparisons. Children with lower-limb pain had a substantially higher BMI (21.37 ± 5.40 vs. 17.63 ± 3.79 kg·m^−2^; *d* = 0.91; *p* < 0.001) and body mass (45.09 ± 20.36 vs. 36.09 ± 15.03 kg; *d* = 0.56; *p* < 0.001), whereas age did not differ (*p* = 0.98) and the difference in height was negligible (*d* = 0.06). BAZ was also higher in children with pain (1.31 ± 1.56 vs. 0.01 ± 1.43; *d* = 0.89). The association with foot posture was of an entirely different order of magnitude from every other variable (χ^2^ = 2630.97, df = 3, *p* < 0.001; Cramér *V* = 0.773): pain prevalence was 1.95% in children with normal feet, 25.73% in high-arched feet, 66.25% in flexible flatfoot and 84.03% in rigid flatfoot. By comparison, the associations with sex (17.58% in boys vs. 14.94% in girls; *V* = 0.035), footwear type (range 13.36–21.32% across the eight categories; *V* = 0.057) and footwear quality (19.43% branded original, 15.69% counterfeit, 60.00% unbranded from only ten children; *V* = 0.065) were statistically detectable but trivially small.

### 3.2. Predictive Performance

**Figure 1:**
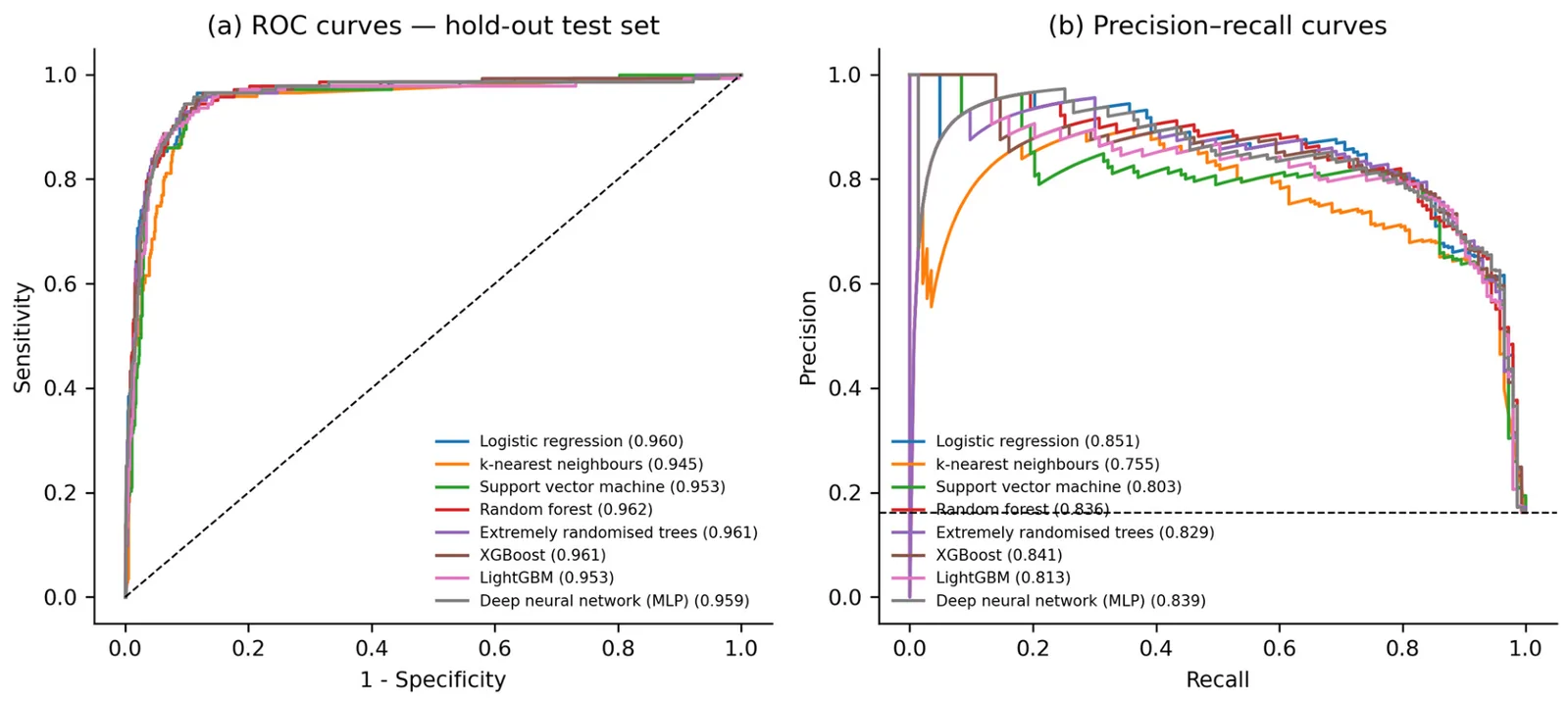
Discrimination in the held-out test partition: (**a**) receiver operating characteristic curves and (**b**) precision–recall curves for all eight classifiers; the dashed line in (**b**) marks the outcome prevalence. Values in parentheses are AUROC and average precision.

Table 2 and Figure 1 summarise performance in the held-out test partition. Discrimination was high for every algorithm, and the spread between algorithms was small (test AUROC 0.945–0.962; AUPRC 0.755–0.851). Extremely randomised trees gave the best cross-validated AUROC (0.968 ± 0.006) and were pre-designated the primary model on that basis; in the test partition this model achieved AUROC 0.961 (95% CI 0.942–0.977), AUPRC 0.829, accuracy 0.916, balanced accuracy 0.910, sensitivity 0.902, specificity 0.919, PPV 0.683, NPV 0.980 and MCC 0.737, corresponding to 679 true negatives, 129 true positives, 60 false positives and 14 false negatives.

The deep neural network performed comparably (test AUROC 0.959; balanced accuracy 0.916) with the highest sensitivity of any model (0.965) at the cost of the lowest PPV (0.582). The bootstrap difference in AUROC between the primary model and penalised logistic regression was 0.0003 (95% CI −0.0075 to 0.0095; *p* = 0.99), i.e., indistinguishable from zero. Interval estimates for all pairwise comparisons overlapped substantially, and no model was superior to logistic regression on any metric by a margin exceeding its CI.

### 3.3. Calibration and Threshold Behaviour

**Figure 2:**
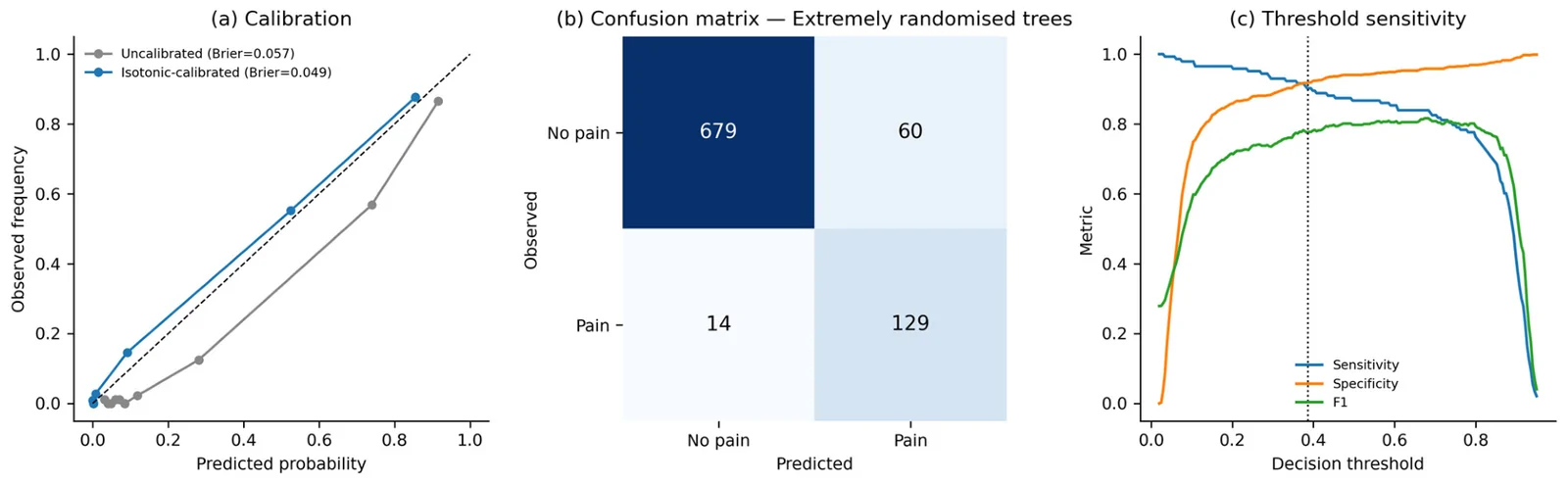
Calibration and threshold behavior of the primary model: (**a**) calibration curves before and after isotonic recalibration, with gray representing the uncalibrated model, blue representing the isotonic-calibrated model, and the black dashed line indicating perfect calibration; (**b**) confusion matrix at the prespecified threshold, with darker blue shading indicating a greater number of observations; and (**c**) sensitivity (blue), specificity (orange), and F1 score (green) across decision thresholds, with the vertical black dotted line indicating the prespecified threshold.

Figure 2 shows calibration, the confusion matrix of the primary model, and the sensitivity of performance to the decision threshold. Class-weighted training produced probabilities that were systematically too extreme; isotonic recalibration improved the Brier score from 0.057 to 0.049 without materially changing discrimination (AUROC 0.960). Residual miscalibration nonetheless remained detectable (Hosmer–Lemeshow χ^2^ = 50.7, df = 5, *p* < 0.001), predominantly in the upper deciles of predicted risk, and this is reported as a limitation rather than resolved. Before recalibration, the calibration intercept was −0.98 (95% CI −1.31 to −0.65) and the calibration slope 0.75 (0.63–0.88); after isotonic recalibration they were 0.40 (95% CI 0.07–0.72) and 0.80 (0.68–0.93), respectively (ideal values 0 and 1). For penalised logistic regression, the corresponding values were −1.28 (95% CI −1.59 to −0.97) and 0.94 (0.80–1.07). Threshold analysis showed the expected trade-off, with balanced accuracy stable across a broad plateau of thresholds between approximately 0.25 and 0.55.

### 3.4. Where the Predictive Information Resides

**Figure 3:**
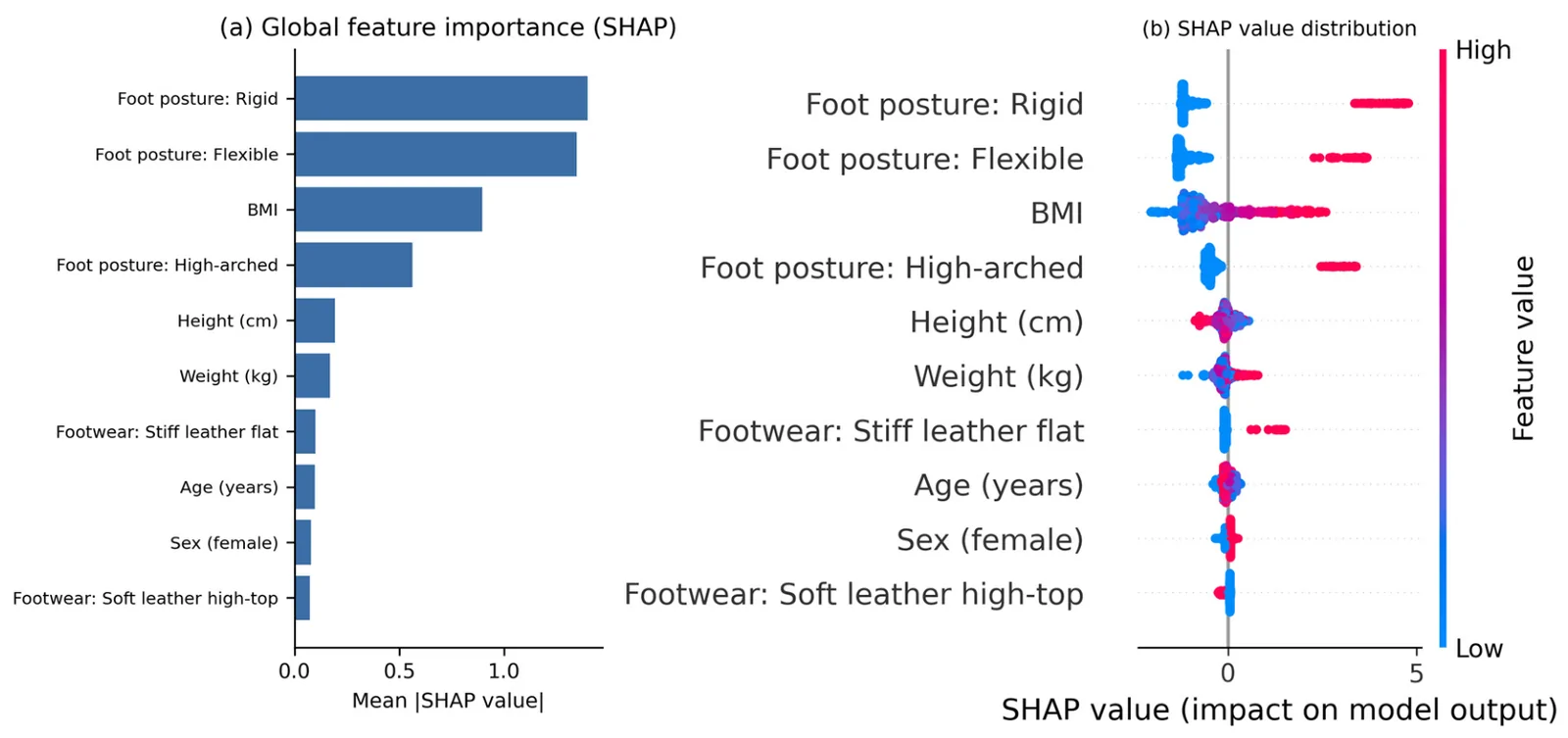
Model explanation for the gradient-boosted classifier: (**a**) mean absolute SHAP values for the ten most influential features; (**b**) distribution of per-child SHAP values, coloured by feature value.

**Figure 4:**
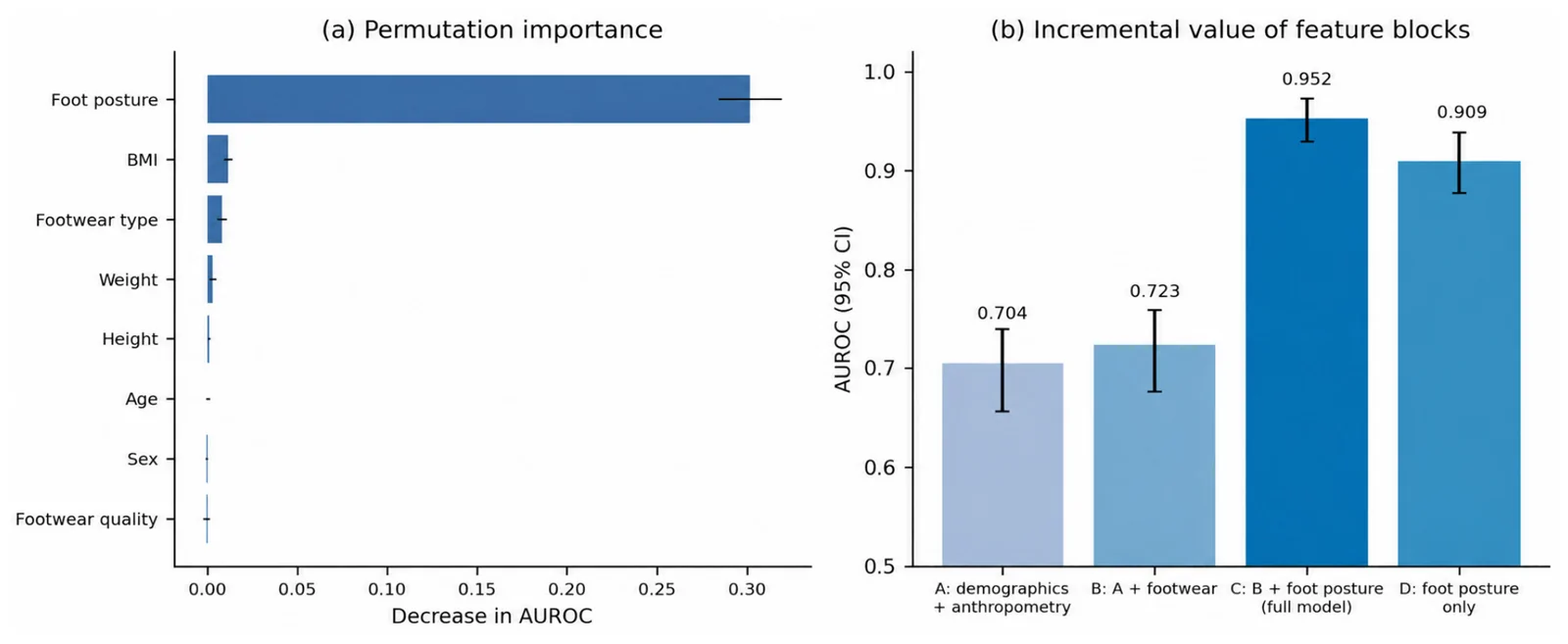
Predictor contributions: (**a**) permutation importance for the primary model (decrease in test AUROC over 30 permutations, error bars = SD); (**b**) AUROC of the four nested predictor blocks with bootstrap 95% confidence intervals.

**Figure 5:**
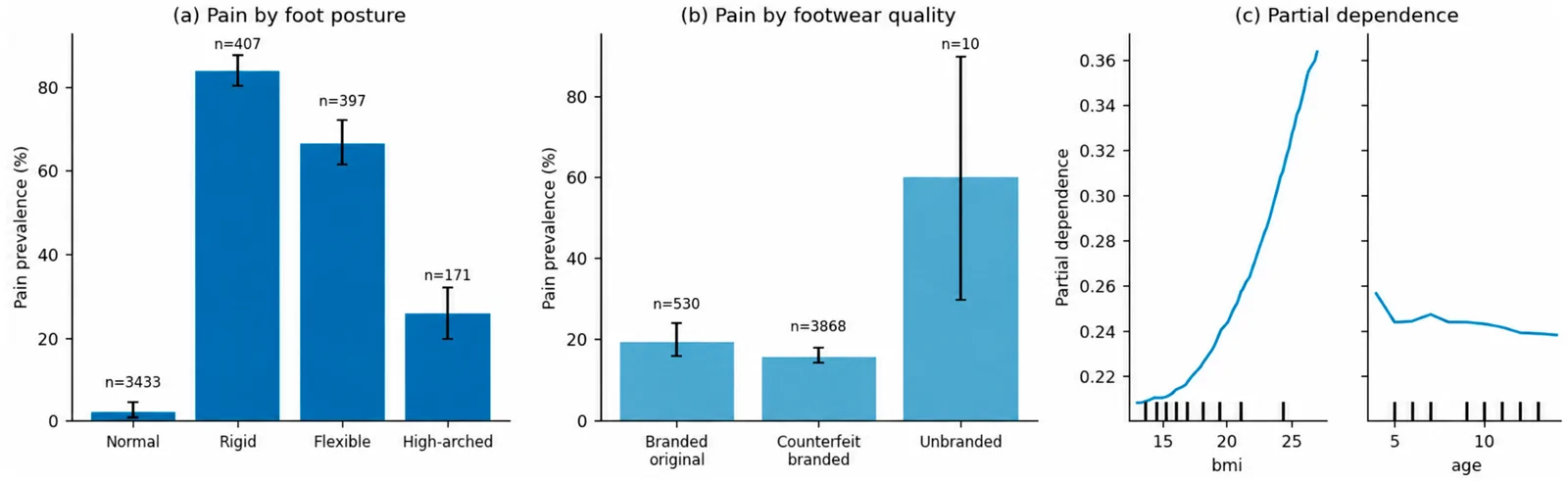
Observed and modelled risk: observed lower-limb pain prevalence by (**a**) foot posture and (**b**) footwear quality with 95% confidence intervals and group sizes; (**c**) partial dependence of predicted risk on BMI and age for the primary model.

Permutation importance and SHAP analysis converged on the same conclusion (Figure 3 and Figure 4a). Permuting foot posture reduced test AUROC by 0.302 ± 0.018, an effect 26 times larger than that of BMI (0.011 ± 0.002) and 38 times larger than that of footwear type (0.008 ± 0.003). Permuting footwear quality (−0.001 ± 0.002), sex (−0.000 ± 0.001), and age (0.000 ± 0.001) produced no detectable loss. In the SHAP decomposition, the indicators for rigid (mean |SHAP| = 1.40) and flexible (1.35) flatfoot dominated, followed by BMI (0.89) and the high-arched indicator (0.56); every footwear-related feature contributed an order of magnitude less, the largest being the stiff leather flat-soled shoe (0.10). The partial-dependence plot (Figure 5c) shows a monotonic and clinically coherent rise in predicted risk with BMI and an essentially flat relationship with age.

The nested models quantified the same structure directly (Table 3, Figure 4b). Demographics and anthropometry alone reached AUROC 0.704 (95% CI 0.657–0.753). Adding both footwear variables raised this to 0.723 (0.676–0.767), an increment of 0.019. Adding foot posture raised it to 0.952 (0.929–0.972)—a gain of 0.230 over the footwear-augmented model (95% CI 0.184–0.275; *p* < 0.001). Foot posture alone already achieved 0.909 (0.877–0.938); the full model exceeded it by only 0.043 (95% CI 0.018–0.070), a small but statistically detectable increment attributable mainly to BMI.

### 3.5. Multivariable Associations

The logistic regression model (*n* = 4408; McFadden pseudo-*R*^2^ = 0.617; likelihood-ratio *p* < 0.001) reproduced the same hierarchy with interpretable effect sizes (Table 4). Relative to a normal foot, the adjusted odds of pain were 312.0-fold higher for rigid flatfoot (95% CI 205.4–474.0), 110.5-fold higher for flexible flatfoot (76.6–159.4), and 38.0-fold higher for a high-arched foot (23.4–61.6). Each unit of BMI multiplied the odds of pain by 1.26 (1.21–1.31). Of the seven footwear-type contrasts, only the stiff leather flat-soled shoe reached significance (OR 6.92, 95% CI 1.94–24.69; *p* = 0.003), and unbranded footwear was associated with markedly increased odds (OR 17.54, 95% CI 2.50–123.01; *p* = 0.004)—an estimate resting on only ten children and correspondingly fragile. Counterfeit branded footwear, worn by 87.7% of the sample, was not associated with pain after adjustment (OR 1.39, 95% CI 0.95–2.03; *p* = 0.086), nor were age (OR 0.95 per year, 0.87–1.03) or female sex (OR 1.18, 0.90–1.55). No predictor category showed complete or quasi-complete separation: every foot-posture category contained children with and without pain, the smallest cell comprising 44 children. Crude Firth-penalised ORs computed from the observed counts were 260.8 (95% CI 183.9–376.2) for rigid flatfoot, 97.7 (71.5–135.2) for flexible flatfoot and 17.4 (11.4–26.4) for high-arched feet, virtually identical to the unpenalised crude estimates (Table 5); the adjusted Firth-penalised ORs were 293.8 (196.3–449.5), 105.5 (74.0–153.0) and 36.9 (22.8–59.7), respectively. The magnitude of these ORs therefore reflects the extreme contrast in prevalence (84.0%, 66.2% and 25.7% vs. 2.0%) rather than sparse-data bias. Because the outcome was common, PRs express the same contrasts on a more intuitive scale: 43.1 (33.8–54.8), 33.9 (26.5–43.5) and 13.2 (9.3–18.7), respectively. The only sparse contrast, unbranded footwear (6 of 10 children with pain), had a crude Firth-penalised OR of 6.0 (1.8–21.9) and an adjusted Firth-penalised OR of 17.5 (2.4–115.2), and remains unstable.

### 3.6. Sensitivity and Secondary Analyses

Excluding the 20 children with implausible BMI values left performance unchanged (*n* = 4388; test AUROC 0.962; AUPRC 0.836). Replacing BMI with BAZ gave a test AUROC of 0.959 (95% CI 0.939–0.975) for the primary model. In the multivariable logistic model the adjusted OR was 1.69 (1.53–1.87) per 1-SD increase in BAZ; the association departed from linearity (spline test *p* = 0.002) and strengthened with age (BAZ × age interaction *p* < 0.001), the OR per SD being 1.21 (1.02–1.42) at 5 years, 1.84 (1.64–2.05) at 10 years and 2.36 (1.98–2.82) at 13 years. Absolute BMI, by contrast, showed no departure from linearity (*p* = 0.95) and only a borderline interaction with age (*p* = 0.054). Deliberately re-introducing the pain-site variable as a predictor produced perfect apparent performance (test AUROC 1.000, accuracy 1.000), confirming that this single variable is sufficient to fabricate a flawless model. In the secondary five-class analysis of pain site among the 716 symptomatic children, cross-validated accuracy was 0.631 against a majority-class baseline of 0.617, with macro-averaged F1 of 0.506 and Cohen κ of 0.343; the model discriminated plantar and anterior-leg pain reasonably but failed almost entirely for ankle pain (*n* = 27). Anatomical localisation of pain is therefore not predictable from these variables, and we report it as a negative finding.

Region-specific analyses are summarised in Table 6. Foot posture remained strongly associated with both outcomes, but the pattern differed by site. For pain confined to the leg (487 cases), foot posture alone reached a test AUROC of 0.957 (0.931–0.978), no lower than the full model (0.953; 0.920–0.977), and pain was reported by 0.4% of children with normal feet against 78.5%, 59.0% and 24.4% of those with rigid flatfoot, flexible flatfoot and high-arched feet. For pain involving the foot or ankle (229 cases), foot posture alone reached 0.833 (0.767–0.895), whereas the full model reached 0.957 (0.931–0.976); the high-arched foot was only weakly associated with this outcome (adjusted OR 3.7, 1.1–12.9; 2.3% vs. 1.5% in normal feet), and BMI carried more information (adjusted OR 1.33 per kg·m^−2^, 1.27–1.40, vs. 1.15, 1.09–1.20, for pain confined to the leg). Firth penalisation changed these adjusted ORs only modestly (rigid and flexible flatfoot: 105.3 and 31.8 for pain involving the foot or ankle; 728.1 and 287.8 for pain confined to the leg; Table 6), so they do not reflect sparse-data bias; the leg-only estimates are nevertheless extreme and imprecise because only 15 of 3381 children with normal feet reported leg-only pain, and the corresponding crude PRs were 177.0 (106.5–294.4) and 133.0 (79.7–222.2).

## 4. Discussion

### 4.1. Principal Findings

In 4408 children, concurrent lower-limb pain was discriminated with high accuracy—but for a reason that reframes rather than supports the current enthusiasm for algorithmic modelling in this field. Essentially all of the predictive signal was carried by a single clinical variable, foot posture, which alone reached an AUROC of 0.909; the remaining seven variables together added 0.043. Anthropometry contributed a modest, biologically coherent increment through BMI, and habitual footwear—the factor most often blamed by parents and most often targeted by commercial claims—contributed almost nothing, raising AUROC by 0.019 when added to a demographic model and being ranked last by both permutation importance and SHAP.

The second principal finding is methodological. Eight algorithms spanning linear, kernel, instance-based, ensemble, and deep-learning families produced test AUROCs within 0.017 of one another, and the difference between the best model and penalised logistic regression was 0.0003 with a CI straddling zero. This is exactly the pattern that Christodoulou and colleagues described in their systematic review of clinical prediction models [40]: when the feature space is low-dimensional, and the informative structure is largely categorical, flexible learners have nothing extra to exploit. Reporting a deep network as the “best model” for such data would misrepresent a tie as a discovery.

### 4.2. Comparison with Existing Literature

The dominance of foot posture is consistent with the clinical literature. Reviews of paediatric pes planus emphasise that most flexible flat feet are physiological and asymptomatic, whereas rigid deformity should raise suspicion of tarsal coalition or other underlying pathology and is far more likely to be painful [1,3,4,5,6]. Our data quantify that intuition in an unusually large sample: 84.0% of children with rigid flatfoot and 66.2% with flexible flatfoot reported pain, against 2.0% of children with normal feet. The intermediate position of the high-arched foot (25.7%) is likewise consistent with cavus deformity being symptomatic in a substantial minority. Two cautions apply. First, foot posture was analysed as four categories derived from a static footprint index and a clinical flexibility test, rather than as a continuous measure or a multi-segment instrument such as the Foot Posture Index, and inter-observer reliability in flatfoot classification is known to be imperfect [7,29]. Second, and more importantly, both the exposure and the outcome were ascertained at the same visit; symptoms mentioned by a child during the visit could still influence the examination, so some of the association may be diagnostic rather than causal. The foot-posture assessors were blinded to pain status and pain location, which limits, but does not exclude, this form of bias, because pain and posture were recorded during the same school visit. Where the examiner is aware of symptoms, borderline feet may be shifted towards a flatfoot or rigid category—a form of review bias documented in diagnostic research [61]—which would inflate both the observed associations and the discrimination. The very large ORs should also be read alongside the PRs (Table 5), which express the same contrasts on a less exaggerated scale.

Most symptomatic children localised their pain to the leg rather than to the foot: pain was confined to the leg in 68.0% and involved the foot or ankle in 32.0%. Posterior-leg pain in children is often labelled “growing pains”; a pronated foot posture has been proposed as a contributor, with in-shoe devices reducing symptoms in single-case experiments [45], although a controlled comparison of 4–6-year-olds found no clinically meaningful difference in foot posture between children with and without such pain [46]. Gastrocnemius–soleus tightness, which was not measured here, offers a further plausible link between flatfoot and posterior-leg symptoms [20,21]. The region-specific analyses sharpen this interpretation: foot posture, including the high-arched foot, was most tightly linked to pain confined to the leg, whereas pain involving the foot or ankle depended more on adiposity and was barely associated with a high arch. Both patterns are compatible with a mechanical contribution of foot posture and body mass to symptoms, but, being cross-sectional, they cannot distinguish cause from concurrent assessment. The outcome is therefore labelled lower-limb pain deliberately, and the results should not be read as applying to pain confined to the foot.

The association with BMI is consistent with previous biomechanical studies in children. Obese children exhibit flatter footprints, thicker medial midfoot fat pads and structurally different arches [11,12,14,15], generate higher plantar pressures during standing and walking [13,17], and show reduced foot sensitivity [17]; higher plantar loading is in turn associated with lower physical activity [16]. An adjusted OR of 1.26 per BMI unit—approximately a 3.5-fold increase in odds across the interquartile range of our sample—is a clinically meaningful effect that is independent of foot posture. Absolute body mass was also higher in children with pain (*d* = 0.56; Table 1), but less markedly than BMI (*d* = 0.91). Because children with and without pain were similar in age and height (*d* = 0.00 and 0.06), the stronger BMI signal indicates that excess mass relative to body size, rather than body mass per se, accompanies pain. This agrees with systematic reviews linking higher BMI to non-specific foot pain [62] and paediatric obesity to flatfoot [63]. Absolute mass remains mechanically relevant because it determines plantar loading [13], but in growing children it can be interpreted only relative to height and age. The association persisted when BMI was replaced by the age- and sex-standardised BMI-for-age z-score and was stronger in older than in younger children, so it is not an artefact of the wide age range.

The near-null footwear findings deserve careful interpretation rather than dismissal. Footwear demonstrably alters paediatric gait [24], incorrectly fitted shoes are associated with foot pain and disorders [25], and habitual shoe use is associated with differences in foot morphology and strike pattern [26,27]. Our data do not contradict that evidence; they show that the crude descriptors available here—a broad shoe-type category and a branding label—capture little of it. Shoe fit, which Buldt and Menz identified as the operative variable and which is incorrectly achieved in a majority of wearers [25], was not measured. Nor were sole stiffness, heel-counter rigidity, wear duration, or shoe age, which are the mechanically relevant properties [28]. The one footwear signal that did survive adjustment, a six- to seven-fold increase in odds for stiff leather flat-soled shoes, is mechanically plausible in the direction predicted by the flexibility literature and merits targeted study with objective shoe characterisation. The very large OR for unbranded footwear rests on ten children and should be regarded as hypothesis-generating only.

### 4.3. Methodological Implications for Machine Learning in Paediatric Foot Health

Published ML studies of foot pathology routinely report accuracies above 0.95 [30,31,32,33,34,35], and analyses of tabular foot-survey data have reported AUROCs approaching 0.99 with near-perfect Matthews coefficients. Our leakage experiment shows how such numbers can arise without any modelling skill: retaining the pain-site variable, whose “no pain” level is a deterministic function of the outcome, yielded an AUROC of exactly 1.000 in the same data that support a defensible ceiling of about 0.96. Kapoor and Narayanan catalogued this failure across 17 fields and 294 papers [42]; foot-health research is not exempt. We would therefore encourage three concrete practices, each of which is already required by TRIPOD+AI and assessed by PROBAST+AI [36,37]: publish the complete predictor list with an explicit statement of why each variable is available before the outcome is known; report the performance of a logistic-regression comparator alongside any complex model; and report calibration, not discrimination alone.

The calibration result illustrates the last point. Our primary model discriminated excellently yet remained imperfectly calibrated even after isotonic recalibration, with predicted risks too extreme in the upper deciles—a direct consequence of class-weighted training. Van Calster and colleagues describe calibration as the Achilles heel of predictive analytics precisely because a well-discriminating but miscalibrated model cannot safely support a “refer if predicted risk exceeds *x*%” rule [39]. Any deployment of a model such as ours would require recalibration in the target population. The calibration intercept and slope (Section 3.3) quantify this: before recalibration, the negative intercepts of the primary model (−0.98) and of class-weighted logistic regression (−1.28) indicate systematic over-estimation of risk, a direct consequence of class weighting; isotonic recalibration reduced this bias but reversed its direction (intercept 0.40), and its slope below 1 (0.80) shows that the tree-based predictions remained too extreme, whereas logistic regression ranked risk with a near-ideal slope (0.94) and would require only an intercept update. Predicted probabilities from the present model should therefore be used, at most, to rank children for clinical examination, not as absolute risks, and no risk threshold derived from these data should be transferred to practice without local recalibration.

### 4.4. Clinical Implications

These findings are addressed primarily to general practitioners, paediatricians and school-health staff who perform first-contact assessment of children with lower-limb complaints, rather than to surgeons; the dataset contains no information on surgical indications or outcomes. For the clinician, the practical message is one of parsimony. In this population, the information that a single school examination provides about which children currently report lower-limb pain is carried by two observations that require no equipment: the clinical foot posture and the child’s weight status. A model built on foot posture alone discriminated children who were symptomatic at the time of examination with an AUROC of 0.909, and adding seven further variables—including everything recorded about footwear—raised this by 0.043. Conversely, the NPV of the full model was 0.980, so a child with a normal foot posture and unremarkable BMI was very unlikely to report lower-limb pain at the time of examination, whereas its PPV of 0.683 means roughly one in three flagged children would not report pain. Because pain and posture were recorded concurrently, this is case-finding among children who already have symptoms, not prediction of who will develop pain; the model has been neither externally nor prospectively validated, and its residual miscalibration (Section 3.3) precludes fixed risk-threshold rules. At most, the findings support including a brief foot-posture inspection and weight-status assessment in routine school or primary-care examinations, so that children with rigid or painful feet are examined further; they do not support allocating treatment. Given that most flexible flat feet in children are asymptomatic and self-limiting [1,3], and that non-surgical intervention for flexible paediatric flatfoot has limited supporting evidence [3], such information should be used only to prioritise clinical assessment, never to justify pre-emptive orthotic prescription.

Parents and school health services may also take note of what was not associated with pain. Within this sample, shoe branding and authenticity—a salient and financially consequential concern for families—showed no independent association once foot posture and BMI were accounted for. Attention and resources are better directed to weight status and to identification of the rigid or painful foot that warrants specialist referral.

### 4.5. Strengths and Limitations

Strengths include the sample size, which comfortably exceeds the events-per-parameter requirements for a model of this dimensionality [41]; a pre-specified, leakage-audited pipeline with all preprocessing inside cross-validation; a genuinely held-out test partition used once; thresholds fixed from training data rather than optimised on the test data; bootstrap interval estimates for all headline metrics and for between-model differences; dual interpretability analyses; and explicit sensitivity and negative-control analyses.

The limitations are substantial and constrain interpretation. The design is cross-sectional, so no causal or temporal claim can be made; foot posture and pain were assessed concurrently. Lower-limb pain was ascertained by report as a binary variable without intensity, duration, frequency, or a validated instrument, and the question referred only to pain after sports activities or prolonged walking, so rest pain was not captured. The pain sites extended beyond the foot, and although grouped region-specific analyses were performed, the small numbers of ankle, plantar-foot and anterior-leg cases precluded site-by-site modelling. Foot posture was available only as four categories derived from the Staheli Arch Index and the Jack test; the continuous index values were not retained, and inter-rater reliability could not be quantified [29]. Although the assessors were blinded to pain status and pain location, both were recorded during the same school visit, so inadvertent disclosure of symptoms by children cannot be completely excluded. The rigid category was defined clinically from the footprint index and the Jack test, without radiographic or subtalar range-of-motion confirmation; therefore, it is not equivalent to structurally rigid flatfoot such as tarsal coalition [1], and its frequency in this sample (9.2%) should be interpreted in this light. Footwear was characterised only by broad type and branding; fit, size adequacy, sole stiffness and duration of wear—the properties the literature identifies as mechanically relevant [25,28]—were unavailable, which very probably attenuates the true footwear effect. Physical activity, sports participation, joint hypermobility, gastrocnemius–soleus flexibility (ankle dorsiflexion range of motion), family history and pubertal status were not recorded and are plausible confounders. Validation was internal only: no temporal or external validation was performed, and the model should not be applied elsewhere without recalibration. Children were nested within multiple schools, but school identifiers were not retained, so the random split ignored clustering; internal validation may therefore be optimistic if school-level factors influenced both foot assessment and pain reporting [64]. Performance was not formally evaluated for fairness across subgroups such as sex or age band, and the model was not registered in a prediction-model registry. Finally, the sample is drawn from a single geographic and cultural setting in which counterfeit footwear predominates (87.7% of children), which limits both generalisability and the statistical power to separate the branded-original and unbranded categories.

### 4.6. Future Directions

Three extensions follow directly. First, foot posture should be replaced by a validated, continuous measure such as the Foot Posture Index or a quantitative arch index, which would permit dose–response modelling rather than four-level contrasts and reduce misclassification [7,23]. Such assessment should be performed by examiners blinded to pain status and with documented inter-rater reliability. Second, footwear should be characterised objectively—internal length and width relative to the foot, sole bending stiffness, heel-counter stability and estimated wear hours—since only then can the null result reported here be interpreted as a property of footwear rather than of its measurement. Third, prospective follow-up of the children with asymptomatic flexible flatfoot would establish whether the model identifies a state or a trajectory, which is the question clinicians actually face. We envisage a school-based cohort in which children are re-examined at 12 and 24 months by the same trained examiners, using the Foot Posture Index [65], ankle dorsiflexion range with the knee extended and flexed (to separate gastrocnemius from soleus tightness), and repeated anthropometry expressed as BAZ; pain would be recorded with an age-appropriate instrument capturing site, frequency, intensity, and activity limitation, and incident pain in initially asymptomatic children would be the primary outcome. Such a design would convert the present diagnostic model into a prognostic one and permit temporal and external validation. Clinically, children identified at a school or primary-care examination—particularly those with rigid flatfoot, persistent pain, restricted subtalar motion or limited ankle dorsiflexion—should be re-assessed by the general practitioner and referred for specialist evaluation if these features persist [1,21], whereas an asymptomatic flexible flatfoot warrants reassurance and periodic review rather than intervention [1,3].

## 5. Conclusions

In a cross-sectional sample of 4408 children aged 4–14 years, concurrent lower-limb pain was discriminated with a test AUROC of approximately 0.96, but this performance rested almost entirely on clinically assessed foot posture, with a modest independent contribution from BMI and essentially none from habitual footwear type or footwear authenticity. Eight algorithms, including a deep neural network, were statistically indistinguishable from penalised logistic regression, and a deliberately leaked model reached a perfect AUROC of 1.000 in the same data—a caution against the near-perfect accuracies frequently reported in this literature. For general practitioners and school-health services, first-contact assessment of children with lower-limb complaints is best directed at foot posture and weight status; for researchers, modelling effort is best directed at transparent predictor auditing, parsimonious comparators and calibrated risk estimates rather than at algorithmic complexity. Because exposure and outcome were measured concurrently, prospective studies are needed to establish whether these factors also predict future pain.

## Figures and Tables

**Table 1:** Characteristics of the analytic sample (*n* = 4408) by lower-limb pain status.

Variable	All (*n* = 4408)	Pain (*n* = 716)	No Pain (*n* = 3692)	Test Statistic	*p*	Effect Size
Age, years	9.51 ± 2.83	9.51 ± 2.95	9.51 ± 2.81	*t* = 0.02	0.981	*d* = 0.00
Height, cm	139.96 ± 18.12	140.87 ± 19.33	139.78 ± 17.88	*t* = 1.39	0.164	*d* = 0.06
Body mass, kg	37.55 ± 16.36	45.09 ± 20.36	36.09 ± 15.03	*t* = 11.25	<0.001	*d* = 0.56
BMI, kg·m^−2^	18.24 ± 4.32	21.37 ± 5.40	17.63 ± 3.79	*t* = 17.69	<0.001	*d* = 0.91
Sex (boys/girls), %	49.4/50.6	17.58/14.94 ^1^	—	χ^2^ = 5.44	0.020	*V* = 0.035
Foot posture, % with pain	—	—	—	χ^2^ = 2630.97	<0.001	*V* = 0.773
Normal (*n* = 3433)	77.9%	1.95%	98.05%			
Rigid flatfoot (*n* = 407)	9.2%	84.03%	15.97%			
Flexible flatfoot (*n* = 397)	9.0%	66.25%	33.75%			
High-arched (*n* = 171)	3.9%	25.73%	74.27%			
Footwear type (8 categories)	—	13.36–21.32% ^2^	—	χ^2^ = 14.48	0.043	*V* = 0.057
Footwear quality	—	—	—	χ^2^ = 18.90	<0.001	*V* = 0.065
Branded original (*n* = 530)	12.0%	19.43%	80.57%			
Counterfeit branded (*n* = 3868)	87.7%	15.69%	84.31%			
Unbranded (*n* = 10)	0.2%	60.00%	40.00%			

Values are mean ± SD or percentage. ^1^ Percentage of boys and girls reporting pain. ^2^ Range of pain prevalence across the eight footwear categories. BMI, body mass index; *d*, Cohen’s *d*; *V*, Cramér’s *V*. Mann–Whitney *U* test *p*-values: age, 0.754; height, 0.048; body mass, <0.001; BMI, <0.001; for height, the Mann–Whitney test (*p* = 0.048) and the Welch test (*p* = 0.164) disagreed, but the difference was negligible in size (*d* = 0.06).

**Table 2:** Performance of eight classifiers in the held-out test partition (*n* = 882; 143 events).

Model	CV AUROC (Mean ± SD)	Test AUROC (95% CI)	AUPRC	Acc.	Bal. Acc.	Sens.	Spec.	PPV	NPV	F1	MCC	Brier
Logistic regression (*C* = 1)	0.965 ± 0.007	0.960 (0.939–0.977)	0.851	0.908	0.911	0.916	0.907	0.655	0.982	0.764	0.724	0.059
k-nearest neighbours (*k* = 35)	0.943 ± 0.012	0.945 (0.923–0.964)	0.755	0.883	0.913	0.958	0.869	0.585	0.991	0.727	0.690	0.077
Support vector machine (RBF)	0.962 ± 0.007	0.953 (0.933–0.970)	0.803	0.904	0.900	0.895	0.905	0.646	0.978	0.751	0.707	0.057
Random forest (500 trees)	0.967 ± 0.007	0.962 (0.943–0.978)	0.836	0.915	0.910	0.902	0.917	0.679	0.980	0.775	0.735	0.059
Extremely randomised trees ^1^	0.968 ± 0.006	0.961 (0.942–0.977)	0.829	0.916	0.910	0.902	0.919	0.683	0.980	0.777	0.737	0.057
XGBoost (300, depth 3, η 0.05)	0.965 ± 0.008	0.961 (0.939–0.976)	0.841	0.912	0.913	0.916	0.911	0.665	0.982	0.771	0.732	0.057
LightGBM (300, 15 leaves)	0.959 ± 0.011	0.953 (0.927–0.973)	0.813	0.891	0.907	0.930	0.884	0.607	0.985	0.735	0.694	0.056
Deep neural network (128-64-32)	0.965 ± 0.008	0.959 (0.936–0.976)	0.839	0.882	0.916	0.965	0.866	0.582	0.992	0.726	0.691	0.050

^1^ Primary model, pre-designated by cross-validated AUROC. Thresholds were fixed from training-fold Youden indices. Acc., accuracy; AUPRC, area under the precision–recall curve; AUROC, area under the receiver operating characteristic curve; CI, confidence interval; CV, cross-validation; MCC, Matthews correlation coefficient; NPV, negative predictive value; PPV, positive predictive value; RBF, radial basis function.

**Table 3:** Incremental value of predictor blocks (gradient-boosting learner, held-out test partition).

Model	Predictors	AUROC (95% CI)	AUPRC	ΔAUROC vs. Full (95% CI)	*p*
A	Sex, age, height, mass, BMI	0.704 (0.657–0.753)	0.334	0.247 (0.198–0.298)	<0.001
B	A + footwear type + footwear quality	0.723 (0.676–0.767)	0.333	0.230 (0.184–0.275)	<0.001
C (full)	B + foot posture	0.952 (0.929–0.972)	0.824	reference	—
D	Foot posture only	0.909 (0.877–0.938)	0.730	0.043 (0.018–0.070)	0.001

Differences and CIs from 2000 bootstrap resamples of the test partition. AUPRC, area under the precision–recall curve; AUROC, area under the receiver operating characteristic curve; BMI, body mass index; CI, confidence interval.

**Table 4:** Adjusted odds ratios for lower-limb pain from multivariable logistic regression (*n* = 4408).

Predictor	Adjusted OR	95% CI	*p*
BMI (per 1 kg·m^−2^)	1.26	1.21–1.31	<0.001
Age (per year)	0.95	0.87–1.03	0.194
Female sex (vs. male)	1.18	0.90–1.55	0.228
Foot posture: rigid flatfoot (vs. normal)	312.00	205.36–474.02	<0.001
Foot posture: flexible flatfoot (vs. normal)	110.46	76.57–159.36	<0.001
Foot posture: high-arched (vs. normal)	37.95	23.39–61.57	<0.001
Footwear: stiff leather flat (vs. canvas high-top)	6.92	1.94–24.69	0.003
Footwear: closed-toe slipper (vs. canvas high-top)	1.79	0.52–6.15	0.354
Footwear: sandal (vs. canvas high-top)	1.85	0.46–7.38	0.386
Footwear quality: counterfeit branded (vs. original)	1.39	0.95–2.03	0.086
Footwear quality: unbranded (vs. original)	17.54	2.50–123.01	0.004

Remaining footwear-type contrasts (leather high-top flat, soft canvas flat, soft leather high-top, medium-stiff leather thick-soled) had odds ratios between 1.07 and 1.32, all *p* > 0.56. BMI, body mass index; CI, confidence interval; OR, odds ratio.

**Table 5:** Crude and Firth-penalised odds ratios and prevalence ratios for the sparse or extreme contrasts.

Contrast	Pain, *n*/*N* (%)	Crude OR (95% CI)	Crude Firth OR (95% CI)	Crude PR (95% CI)	Adjusted Firth OR (95% CI) ^1^
Rigid flatfoot vs. normal	342/407 (84.0) vs. 67/3433 (2.0)	264.3 (184.6–378.5)	260.8 (183.9–376.2)	43.1 (33.8–54.8)	293.8 (196.3–449.5)
Flexible flatfoot vs. normal	263/397 (66.2) vs. 67/3433 (2.0)	98.6 (71.7–135.7)	97.7 (71.5–135.2)	33.9 (26.5–43.5)	105.5 (74.0–153.0)
High-arched vs. normal	44/171 (25.7) vs. 67/3433 (2.0)	17.4 (11.4–26.5)	17.4 (11.4–26.4)	13.2 (9.3–18.7)	36.9 (22.8–59.7)
Unbranded vs. branded original	6/10 (60.0) vs. 103/530 (19.4)	6.2 (1.7–22.4)	6.0 (1.8–21.9)	3.1 (1.8–5.3)	17.5 (2.4–115.2)

Crude estimates were computed from the observed counts (Table 1); Firth-penalised estimates are given with profile-penalised likelihood 95% CIs and PRs with log-method 95% CIs. ^1^ Adjusted for age, BMI, sex, foot posture, footwear type and footwear quality, as in Table 4. CI, confidence interval; OR, odds ratio; PR, prevalence ratio.

**Table 6:** Region-specific analyses: discrimination and adjusted foot-posture odds ratios by pain location.

Outcome Definition	Cases/Controls	AUROC, Full Model (95% CI) ^1^	AUROC, Foot Posture Only (95% CI) ^1^	Adjusted OR, Rigid Flatfoot (95% CI) ^2^	Adjusted OR, Flexible Flatfoot (95% CI) ^2^
Any lower-limb pain (primary)	716/3692	0.950 (0.926–0.970)	0.909 (0.877–0.938)	312.0 (205.4–474.0)	110.5 (76.6–159.4)
Pain involving the foot or ankle	229/3692	0.957 (0.931–0.976)	0.833 (0.767–0.895)	114.2 (66.5–196.1)	33.7 (20.5–55.4)
Pain confined to the leg	487/3692	0.953 (0.920–0.977)	0.957 (0.931–0.978)	807.7 (441.8–1476.6)	313.6 (177.7–553.2)

^1^ Histogram-based gradient boosting (default settings) fitted to the original training partition for each outcome and evaluated in the held-out test partition (cases/controls in the test partition: 143/739, 55/739 and 88/739); 95% CIs from 2000 bootstrap resamples. ^2^ Multivariable logistic regression with the covariates of Table 4; the primary-outcome ORs are those of Table 4. Firth-penalised adjusted ORs (rigid; flexible flatfoot): foot or ankle, 105.3 (62.7–181.9) and 31.8 (19.6–52.4); leg only, 728.1 (415.7–1360.6) and 287.8 (170.3–520.3). Crude PRs: foot or ankle, 40.4 (30.1–54.3) and 22.6 (16.2–31.4); leg only, 177.0 (106.5–294.4) and 133.0 (79.7–222.2). Foot or ankle: plantar foot, ankle, or posterior leg with medial foot; leg only: posterior or anterior leg. AUROC, area under the receiver operating characteristic curve; CI, confidence interval; OR, odds ratio; PR, prevalence ratio.

## Data Availability

The de-identified data and analysis code supporting the findings may be made available by the corresponding author upon reasonable request, subject to ethical, legal, and institutional data-governance restrictions.

## Supplementary Materials

The following supporting information can be downloaded at https://www.mdpi.com/article/10.3390/life16101639/s1: Table S1: TRIPOD+AI checklist; Table S2: STROBE checklist for cross-sectional studies.

## Abbreviations

The following abbreviations are used in this manuscript:

AI	Artificial intelligence
AUPRC	Area under the precision–recall curve
AUROC	Area under the receiver operating characteristic curve
BAZ	Body mass index-for-age z-score
BMI	Body mass index
CI	Confidence interval
CV	Cross-validation
DNN	Deep neural network (multilayer perceptron)
MCC	Matthews correlation coefficient
ML	Machine learning
NPV	Negative predictive value
OR	Odds ratio
PPV	Positive predictive value
PR	Prevalence ratio
SHAP	SHapley Additive exPlanations
STROBE	Strengthening the Reporting of Observational Studies in Epidemiology
TRIPOD+AI	Transparent Reporting of a multivariable prediction model for Individual Prognosis Or Diagnosis + Artificial Intelligence

## References

[1] Carr J.B., Yang S., Lather L.A. (2016). Pediatric pes planus: A state-of-the-art review. Pediatrics.

[2] Pfeiffer M., Kotz R., Ledl T., Hauser G., Sluga M. (2006). Prevalence of flat foot in preschool-aged children. Pediatrics.

[3] Evans A.M., Rome K. (2011). A Cochrane review of the evidence for non-surgical interventions for flexible pediatric flat feet. Eur. J. Phys. Rehabil. Med..

[4] Michaudet C., Edenfield K.M., Nicolette G.W., Carek P.J. (2018). Foot and ankle conditions: Pes planus. FP Essent..

[5] Yagerman S.E., Cross M.B., Positano R., Doyle S.M. (2011). Evaluation and treatment of symptomatic pes planus. Curr. Opin. Pediatr..

[6] Ueki Y., Sakuma E., Wada I. (2019). Pathology and management of flexible flat foot in children. J. Orthop. Sci..

[7] Boryczka-Trefler A., Kalinowska M., Szczerbik E., Stępowska J., Łukaszewska A., Syczewska M. (2023). How to define pediatric flatfoot: Comparison of 2 methods: Foot posture in static and dynamic conditions in children 5 to 9 years old. Foot Ankle Spec..

[8] Shih Y.F., Chen C.Y., Chen W.Y., Lin H.C., Su F.C. (2012). Lower extremity kinematics in children with and without flexible flatfoot: A comparative study. BMC Musculoskelet. Disord..

[9] Lee J.H., Sung I.Y., Yoo J.Y. (2009). Clinical or radiologic measurements and 3-D gait analysis in children with pes planus. Pediatr. Int..

[10] Kim M.H., Cha S., Choi J.E., Jeon M., Choi J.Y., Yang S.S. (2023). Relation of flatfoot severity with flexibility and isometric strength of the foot and trunk extensors in children. Children.

[11] Riddiford-Harland D.L., Steele J.R., Storlien L.H. (2000). Does obesity influence foot structure in prepubescent children?. Int. J. Obes. Relat. Metab. Disord..

[12] Dowling A.M., Steele J.R., Baur L.A. (2001). Does obesity influence foot structure and plantar pressure patterns in prepubescent children?. Int. J. Obes. Relat. Metab. Disord..

[13] Dowling A.M., Steele J.R., Baur L.A. (2004). What are the effects of obesity in children on plantar pressure distributions?. Int. J. Obes..

[14] Mickle K.J., Steele J.R., Munro B.J. (2006). The feet of overweight and obese young children: Are they flat or fat?. Obesity.

[15] Mauch M., Grau S., Krauss I., Maiwald C., Horstmann T. (2008). Foot morphology of normal, underweight and overweight children. Int. J. Obes..

[16] Riddiford-Harland D.L., Steele J.R., Cliff D.P., Okely A.D., Morgan P.J., Jones R.A., Baur L.A. (2015). Lower activity levels are related to higher plantar pressures in overweight children. Med. Sci. Sports Exerc..

[17] da Rocha E.S., Bratz D.T.K., Gubert L.C., de David A., Carpes F.P. (2014). Obese children experience higher plantar pressure and lower foot sensitivity than non-obese. Clin. Biomech..

[18] Jankowicz-Szymanska A., Mikolajczyk E., Wodka K. (2017). Correlations among foot arching, ankle dorsiflexion range of motion, and obesity level in primary school children. J. Am. Podiatr. Med. Assoc..

[19] Sadeghi-Demneh E., Azadinia F., Jafarian F., Shamsi F., Melvin J.M.A., Jafarpishe M., Rezaeian Z. (2016). Flatfoot and obesity in school-age children: A cross-sectional study. Clin. Obes..

[20] Mosca V.S. (2010). Flexible flatfoot in children and adolescents. J. Child. Orthop..

[21] Harris E.J., Vanore J.V., Thomas J.L., Kravitz S.R., Mendelson S.A., Mendicino R.W., Silvani S.H., Gassen S.C. (2004). Diagnosis and treatment of pediatric flatfoot. J. Foot Ankle Surg..

[22] Müller S., Carlsohn A., Müller J., Baur H., Mayer F. (2012). Static and dynamic foot characteristics in children aged 1–13 years: A cross-sectional study. Gait Posture.

[23] Szczepanowska-Wołowiec B., Sztandera P., Kotela I., Żak M. (2021). Assessment of the foot’s longitudinal arch by different indicators and their correlation with the foot loading paradigm in school-aged children: A cross-sectional study. Int. J. Environ. Res. Public Health.

[24] Wegener C., Hunt A.E., Vanwanseele B., Burns J., Smith R.M. (2011). Effect of children’s shoes on gait: A systematic review and meta-analysis. J. Foot Ankle Res..

[25] Buldt A.K., Menz H.B. (2018). Incorrectly fitted footwear, foot pain and foot disorders: A systematic search and narrative review of the literature. J. Foot Ankle Res..

[26] Rao U.B., Joseph B. (1992). The influence of footwear on the prevalence of flat foot. A survey of 2300 children. J. Bone Jt. Surg. Br..

[27] Hollander K., de Villiers J.E., Venter R., Sehner S., Wegscheider K., Braumann K.M., Zech A. (2018). Foot strike patterns differ between children and adolescents growing up barefoot vs. shod. Int. J. Sports Med..

[28] Hill M., Healy A., Chockalingam N. (2019). Key concepts in children’s footwear research: A scoping review focusing on therapeutic footwear. J. Foot Ankle Res..

[29] Yildiz K., Cetin T. (2022). Interobserver reliability in the radiological evaluation of flatfoot (pes planus) deformity: A cross-sectional study. J. Foot Ankle Surg..

[30] Zhao Y., Zhou J., Qiu F., Liao X., Jiang J., Chen H., Lin X., Hu Y., He J., Chen J. (2023). A deep learning method for foot-type classification using plantar pressure images. Front. Bioeng. Biotechnol..

[31] Gül Y., Yaman S., Avcı D., Çilengir A.H., Balaban M., Güler H. (2023). A novel deep transfer learning-based approach for automated pes planus diagnosis using X-ray image. Diagnostics.

[32] Doğan K., Selçuk T., Yılmaz A. (2024). A novel model based on CNN–ViT fusion and ensemble learning for the automatic detection of pes planus. J. Clin. Med..

[33] Alsaidi F.A., Moria K.M. (2023). Flatfeet severity-level detection based on alignment measuring. Sensors.

[34] Ryu S.M., Shin K., Shin S.W., Lee S., Kim N. (2022). Enhancement of evaluating flatfoot on a weight-bearing lateral radiograph of the foot with U-Net based semantic segmentation on the long axis of tarsal and metatarsal bones in an active learning manner. Comput. Biol. Med..

[35] Danacı Ç., Avcı D., Tuncer S.A. (2025). Diagnosis of pes planus from X-ray images: Enhanced feature selection with deep learning and machine learning techniques. Biomed. Signal Process. Control.

[36] Collins G.S., Moons K.G.M., Dhiman P., Riley R.D., Beam A.L., Van Calster B., Ghassemi M., Liu X., Reitsma J.B., van Smeden M. (2024). TRIPOD+AI statement: Updated guidance for reporting clinical prediction models that use regression or machine learning methods. BMJ.

[37] Moons K.G.M., Damen J.A.A., Kaul T., Hooft L., Andaur Navarro C., Dhiman P., Beam A.L., Van Calster B., Celi L.A., Denaxas S. (2025). PROBAST+AI: An updated quality, risk of bias, and applicability assessment tool for prediction models using regression or artificial intelligence methods. BMJ.

[38] van Smeden M., Reitsma J.B., Riley R.D., Collins G.S., Moons K.G.M. (2021). Clinical prediction models: Diagnosis versus prognosis. J. Clin. Epidemiol..

[39] Van Calster B., McLernon D.J., van Smeden M., Wynants L., Steyerberg E.W. (2019). Calibration: The Achilles heel of predictive analytics. BMC Med..

[40] Christodoulou E., Ma J., Collins G.S., Steyerberg E.W., Verbakel J.Y., Van Calster B. (2019). A systematic review shows no performance benefit of machine learning over logistic regression for clinical prediction models. J. Clin. Epidemiol..

[41] Riley R.D., Ensor J., Snell K.I.E., Harrell F.E., Martin G.P., Reitsma J.B., Moons K.G.M., Collins G., van Smeden M. (2020). Calculating the sample size required for developing a clinical prediction model. BMJ.

[42] Kapoor S., Narayanan A. (2023). Leakage and the reproducibility crisis in machine-learning-based science. Patterns.

[43] Staheli L.T., Chew D.E., Corbett M. (1987). The longitudinal arch: A survey of eight hundred and eighty-two feet in normal children and adults. J. Bone Jt. Surg. Am..

[44] Jack E.A. (1953). Naviculo-cuneiform fusion in the treatment of flat foot. J. Bone Jt. Surg. Br..

[45] Evans A.M. (2003). Relationship between “growing pains” and foot posture in children: Single-case experimental designs in clinical practice. J. Am. Podiatr. Med. Assoc..

[46] Evans A.M., Scutter S.D. (2007). Are foot posture and functional health different in children with growing pains?. Pediatr. Int..

[47] de Onis M., WHO Multicentre Growth Reference Study Group (2006). WHO Child Growth Standards based on length/height, weight and age. Acta Paediatr. Suppl..

[48] de Onis M., Onyango A.W., Borghi E., Siyam A., Nishida C., Siekmann J. (2007). Development of a WHO growth reference for school-aged children and adolescents. Bull. World Health Organ..

[49] World Health Organization (2009). WHO AnthroPlus for Personal Computers Manual: Software for Assessing Growth of the World’s Children and Adolescents.

[50] Fagerland M.W. (2012). t-tests, non-parametric tests, and large studies—A paradox of statistical practice?. BMC Med. Res. Methodol..

[51] Greenland S., Mansournia M.A., Altman D.G. (2016). Sparse data bias: A problem hiding in plain sight. BMJ.

[52] Firth D. (1993). Bias reduction of maximum likelihood estimates. Biometrika.

[53] Heinze G., Schemper M. (2002). A solution to the problem of separation in logistic regression. Stat. Med..

[54] Zhang J., Yu K.F. (1998). What’s the relative risk? A method of correcting the odds ratio in cohort studies of common outcomes. JAMA.

[55] Breiman L. (2001). Random forests. Mach. Learn..

[56] Chen T., Guestrin C. (2016). XGBoost: A scalable tree boosting system. Proceedings of the 22nd ACM SIGKDD International Conference on Knowledge Discovery and Data Mining, San Francisco, CA, USA, 13–17 August 2016.

[57] Ke G., Meng Q., Finley T., Wang T., Chen W., Ma W., Ye Q., Liu T.Y. (2017). LightGBM: A highly efficient gradient boosting decision tree. Advances in Neural Information Processing Systems 30.

[58] Van Calster B., Nieboer D., Vergouwe Y., De Cock B., Pencina M.J., Steyerberg E.W. (2016). A calibration hierarchy for risk models was defined: From utopia to empirical data. J. Clin. Epidemiol..

[59] Lundberg S.M., Lee S.I. (2017). A unified approach to interpreting model predictions. Advances in Neural Information Processing Systems 30.

[60] Pedregosa F., Varoquaux G., Gramfort A., Michel V., Thirion B., Grisel O., Blondel M., Prettenhofer P., Weiss R., Dubourg V. (2011). Scikit-learn: Machine learning in Python. J. Mach. Learn. Res..

[61] Whiting P., Rutjes A.W.S., Reitsma J.B., Glas A.S., Bossuyt P.M.M., Kleijnen J. (2004). Sources of variation and bias in studies of diagnostic accuracy: A systematic review. Ann. Intern. Med..

[62] Butterworth P.A., Landorf K.B., Smith S.E., Menz H.B. (2012). The association between body mass index and musculoskeletal foot disorders: A systematic review. Obes. Rev..

[63] Stolzman S., Irby M.B., Callahan A.B., Skelton J.A. (2015). Pes planus and paediatric obesity: A systematic review of the literature. Clin. Obes..

[64] Bouwmeester W., Twisk J.W.R., Kappen T.H., van Klei W.A., Moons K.G.M., Vergouwe Y. (2013). Prediction models for clustered data: Comparison of a random intercept and standard regression model. BMC Med. Res. Methodol..

[65] Redmond A.C., Crosbie J., Ouvrier R.A. (2006). Development and validation of a novel rating system for scoring standing foot posture: The Foot Posture Index. Clin. Biomech..

