# Supplementary material for: Foot Posture Outweighs Habitual Footwear in Discriminating Concurrent Lower-Limb Pain in Children: A Cross-Sectional Interpretable Machine-Learning Study
Source: Life (Basel). 2026 Sep 30;16(10):1639. doi: 10.3390/life16101639 (PMC13706620; doi:10.3390/life16101639)
Supplement: Supplementary file 1 [file life-16-01639-s001.zip › life-4566540-supplementary.pdf]

Supplementary Materials

Foot Posture Outweighs Habitual Footwear in Discriminating Concurrent Lower-Limb Pain in Children: A Cross-Sectional Interpretable Machine-Learning Study

Table S1. TRIPOD+AI checklist

Section/topic	Item	D;E	Checklist item	Location in manuscript
Title	1	D;E	Identify the study as developing or evaluating the performance of a multivariable prediction model, the target population, and the outcome to be predicted	Title
Abstract	2	D;E	See TRIPOD+AI for Abstracts checklist	Abstract
Background	3a	D;E	Explain the healthcare context (including whether diagnostic or prognostic) and rationale for developing or evaluating the prediction model, including references to existing models	Section 1 (paragraphs 1–3); diagnostic nature stated in Sections 1 and 2.5
Background	3b	D;E	Describe the target population and the intended purpose of the prediction model in the context of the care pathway, including its intended users (e.g., healthcare professionals, patients, public)	Sections 1 and 4.4
Background	3c	D;E	Describe any known health inequalities between sociodemographic groups	Not reported
Objectives	4	D;E	Specify the study objectives, including whether the study describes the development or validation of a prediction model (or both)	Section 1 (final paragraph)
Data	5a	D;E	Describe the sources of data separately for the development and evaluation datasets (e.g., randomised trial, cohort, routine care or registry data), the rationale for using these data, and representativeness of the data	Section 2.1; representativeness in Section 4.5
Data	5b	D;E	Specify the dates of the collected participant data, including start and end of participant accrual; and, if applicable, end of follow-up	Not reported
Participants	6a	D;E	Specify key elements of the study setting (e.g., primary care, secondary care, general population) including the number and location of centres	Section 2.1 (school-based setting, Çanakkale city centre and Ezine district; exact number of institutions not retained)
Participants	6b	D;E	Describe the eligibility criteria for study participants	Section 2.1
Participants	6c	D;E	Give details of any treatments received, and how they were handled during model development or evaluation, if relevant	Not applicable
Data preparation	7	D;E	Describe any data pre-processing and quality checking, including whether this was similar across relevant sociodemographic groups	Sections 2.3 and 2.5
Outcome	8a	D;E	Clearly define the outcome that is being predicted and the time horizon, including how and when assessed, the rationale for choosing this outcome, and whether the method of outcome assessment is consistent across sociodemographic groups	Section 2.2 (concurrent outcome; Section 2.5)
Outcome	8b	D;E	If outcome assessment requires subjective interpretation, describe the qualifications and demographic characteristics of the outcome assessors	Not applicable (self-reported outcome)
Outcome	8c	D;E	Report any actions to blind assessment of the outcome to be predicted	Not applicable (self-reported outcome); blinding of predictor assessment in Section 2.2
Predictors	9a	D	Describe the choice of initial predictors (e.g., literature, previous models, all available predictors) and any pre-selection of predictors before model building	Sections 2.2 and 2.5 (all available variables; pain site excluded)

Predictors	9b	D;E	Clearly define all predictors, including how and when they were measured (and any actions to blind assessment of predictors for the outcome and other predictors)	Sections 2.1 and 2.2
Predictors	9c	D;E	If predictor measurement requires subjective interpretation, describe the qualifications and demographic characteristics of the predictor assessors	Section 2.2
Sample size	10	D;E	Explain how the study size was arrived at (separately for development and evaluation), and justify that the study size was sufficient to answer the research question. Include details of any sample size calculation	Sections 2.1, 2.3 and 2.5 (all eligible records; no formal calculation); adequacy discussed in Section 4.5
Missing data	11	D;E	Describe how missing data were handled. Provide reasons for omitting any data	Section 2.3 (no missing values; exclusions with reasons)
Analytical methods	12a	D	Describe how the data were used (e.g., for development and evaluation of model performance) in the analysis, including whether the data were partitioned, considering any sample size requirements	Section 2.5
Analytical methods	12b	D	Depending on the type of model, describe how predictors were handled in the analyses (functional form, rescaling, transformation, or any standardisation)	Sections 2.4 and 2.5
Analytical methods	12c	D	Specify the type of model, rationale, all model-building steps, including any hyperparameter tuning, and method for internal validation	Section 2.5; Table 2
Analytical methods	12d	D;E	Describe if and how any heterogeneity in estimates of model parameter values and model performance was handled and quantified across clusters (e.g., hospitals, countries). See TRIPOD-Cluster for additional considerations	Not examined; reason given in Sections 2.1 and 4.5
Analytical methods	12e	D;E	Specify all measures and plots used (and their rationale) to evaluate model performance (e.g., discrimination, calibration, clinical utility) and, if relevant, to compare multiple models	Section 2.5
Analytical methods	12f	E	Describe any model updating (e.g., recalibration) arising from the model evaluation, either overall or for particular sociodemographic groups or settings	Section 2.5 (isotonic recalibration within the training partition)
Analytical methods	12g	E	For model evaluation, describe how the model predictions were calculated (e.g., formula, code, object, application programming interface)	Section 2.7
Class imbalance	13	D;E	If class imbalance methods were used, state why and how this was done, and any subsequent methods to recalibrate the model or the model predictions	Section 2.5 (class weighting; isotonic recalibration)
Fairness	14	D;E	Describe any approaches that were used to address model fairness and their rationale	Not performed; stated in Section 4.5
Model output	15	D	Specify the output of the prediction model (e.g., probabilities, classification). Provide details and rationale for any classification and how the thresholds were identified	Section 2.5
Development versus evaluation	16	D;E	Identify any differences between the development and evaluation data in healthcare setting, eligibility criteria, outcome, and predictors	Section 2.5 (random stratified split of a single dataset; no differences)
Ethical approval	17	D;E	Name the institutional research board or ethics committee that approved the study and describe the participant-informed consent or the ethics committee waiver of informed consent	Section 2.1; Institutional Review Board and Informed Consent Statements
Funding	18a	D;E	Give the source of funding and the role of the funders for the present study	Funding statement
Conflicts of interest	18b	D;E	Declare any conflicts of interest and financial disclosures for all authors	Conflicts of Interest statement
Protocol	18c	D;E	Indicate where the study protocol can be accessed or state that a protocol was not prepared	Not reported
Registration	18d	D;E	Provide registration information for the study, including register name and registration number, or state that the study was not registered	Section 4.5 (not registered in a prediction-model registry); Informed Consent Statement
Data sharing	18e	D;E	Provide details of the availability of the study data	Data Availability Statement
Code sharing	18f	D;E	Provide details of the availability of the analytical code	Section 2.7; Data Availability Statement

Patient & public involvement	19	D;E	Provide details of any patient and public involvement during the design, conduct, reporting, interpretation, or dissemination of the study or state no involvement	Not reported
Participants	20a	D;E	Describe the flow of participants through the study, including the number of participants with and without the outcome and, if applicable, a summary of the follow-up time. A diagram may be helpful	Sections 2.3 and 3.1
Participants	20b	D;E	Report the characteristics overall and, where applicable, for each data source or setting, including the key dates, key predictors (including demographics), treatments received, sample size, number of outcome events, follow-up time, and amount of missing data. A table may be helpful. Report any differences across key demographic groups	Section 3.1; Table 1 (dates not reported)
Participants	20c	E	For model evaluation, show a comparison with the development data of the distribution of important predictors (demographics, predictors, and outcome)	Not applicable (internal validation within one dataset; stratified split, Section 2.5)
Model development	21	D;E	Specify the number of participants and outcome events in each analysis (e.g., for model development, hyperparameter tuning, model evaluation)	Sections 2.5 and 3.1; Tables 2, 3 and 6
Model specification	22	D	Provide details of the full prediction model (e.g., formula, code, object, API) to allow predictions in new individuals and to enable third-party evaluation and implementation, including any restrictions to access or re-use (e.g., freely available, proprietary)	Table 2 (hyperparameters); Table 4 (logistic model); code available on request (Section 2.7)
Model performance	23a	D;E	Report model performance estimates with confidence intervals, including for any key subgroups (e.g., sociodemographic). Consider plots to aid presentation	Sections 3.2–3.4 and 3.6; Tables 2, 3 and 6; Figures 1, 2 and 4 (sociodemographic subgroups not reported; Section 4.5)
Model performance	23b	D;E	If examined, report results of any heterogeneity in model performance across clusters. See TRIPOD Cluster for additional details	Not examined (Section 4.5)
Model updating	24	E	Report the results from any model updating, including the updated model and subsequent performance	Section 3.3 (isotonic recalibration)
Interpretation	25	D;E	Give an overall interpretation of the main results, including issues of fairness in the context of the objectives and previous studies	Sections 4.1–4.4
Limitations	26	D;E	Discuss any limitations of the study (such as a non-representative sample, sample size, overfitting, missing data) and their effects on any biases, statistical uncertainty, and generalizability	Section 4.5
Usability of the model	27a	D	Describe how poor quality or unavailable input data (e.g., predictor values) should be assessed and handled when implementing the prediction model	Not reported
Usability of the model	27b	D	Discuss whether users will be required to interact in the handling of the input data or use of the model, and what level of expertise is required of users	Section 4.4
Usability of the model	27c	D;E	Discuss any next steps for future research, with a specific view to applicability and generalizability of the model	Section 4.6

Table S2. STROBE checklist for cross-sectional studies (von Elm et al., Lancet 2007;370:1453–1457); item wording abbreviated. Locations refer to section, table and figure numbers of the revised manuscript.

Section/topic	Item	Recommendation	Location in manuscript
Title and abstract	1a	Indicate the study's design with a commonly used term in the title or the abstract	Title; Abstract
Title and abstract	1b	Provide an informative and balanced summary of what was done and what was found	Abstract
Background/rationale	2	Explain the scientific background and rationale	Section 1
Objectives	3	State specific objectives, including any prespecified hypotheses	Section 1 (final paragraph)
Study design	4	Present key elements of study design early in the paper	Section 2.1
Setting	5	Describe the setting, locations and relevant dates, including periods of recruitment, exposure, follow-up and data collection	Section 2.1 (setting and locations; dates not reported)
Participants	6a	Give the eligibility criteria, and the sources and methods of selection of participants	Section 2.1
Variables	7	Clearly define all outcomes, exposures, predictors, potential confounders and effect modifiers	Section 2.2
Data sources/measurement	8	For each variable of interest, give sources of data and details of methods of assessment	Sections 2.1 and 2.2
Bias	9	Describe any efforts to address potential sources of bias	Sections 2.2, 2.3 and 2.5; discussed in Sections 4.2 and 4.5
Study size	10	Explain how the study size was arrived at	Sections 2.1 and 2.3
Quantitative variables	11	Explain how quantitative variables were handled in the analyses	Sections 2.3 and 2.4
Statistical methods	12a	Describe all statistical methods, including those used to control for confounding	Section 2.4
Statistical methods	12b	Describe any methods used to examine subgroups and interactions	Sections 2.4 and 2.6
Statistical methods	12c	Explain how missing data were addressed	Section 2.3
Statistical methods	12d	If applicable, describe analytical methods taking account of sampling strategy	Not applied; reason given in Sections 2.1 and 4.5
Statistical methods	12e	Describe any sensitivity analyses	Section 2.6
Participants	13a	Report numbers of individuals at each stage of study	Sections 2.3 and 3.1
Participants	13b	Give reasons for non-participation at each stage	Section 2.3 (excluded records)
Participants	13c	Consider use of a flow diagram	Not provided (numbers given in Sections 2.3 and 3.1)
Descriptive data	14a	Give characteristics of study participants and information on exposures and potential confounders	Section 3.1; Table 1
Descriptive data	14b	Indicate number of participants with missing data for each variable of interest	Section 2.3 (no missing values)
Outcome data	15	Report numbers of outcome events or summary measures	Section 3.1
Main results	16a	Give unadjusted estimates and, if applicable, confounder-adjusted estimates and their precision; make clear which confounders were adjusted for and why	Section 3.5; Tables 1, 4 and 5
Main results	16b	Report category boundaries when continuous variables were categorised	Sections 2.2 and 2.3
Main results	16c	If relevant, consider translating estimates of relative risk into absolute risk for a meaningful time period	Section 3.1 (prevalences); Table 5 (prevalence ratios)
Other analyses	17	Report other analyses done, e.g., analyses of subgroups and interactions, and sensitivity analyses	Section 3.6; Table 6
Key results	18	Summarise key results with reference to study objectives	Section 4.1
Limitations	19	Discuss limitations of the study, taking into account sources of potential bias or imprecision	Section 4.5
Interpretation	20	Give a cautious overall interpretation of results considering objectives, limitations, multiplicity of analyses, results from similar studies and other relevant evidence	Sections 4.2–4.4
Generalisability	21	Discuss the generalisability (external validity) of the study results	Section 4.5

Funding	22	Give the source of funding and the role of the funders for the present study and, if applicable, for the original study on which the present article is based	Funding statement
